# Supplementary material for: Characteristics of the complete mitochondrial genome of Suhpalacsa longialata (Neuroptera, Ascalaphidae) and its phylogenetic implications
Source: PeerJ. 2018 Nov 14;6:e5914. doi: 10.7717/peerj.5914 (PMC6240338; doi:10.7717/peerj.5914)
Supplement: Supplemental Information 1 — All universal primers were modified according to Simon et al. (2006), Zhang et al. (2008) and Zhang et al. (2018) by comparing to known mayfly mitochondrial genomes. The orientation of primers is as shown in Fig. 1. [file peerj-06-5914-s001.docx]

**Table S1 Universal and specific primers used to amplify the mitochondrial genome of *S. longialata***

| Primers | Number | Primer name | Sequence (5’-3’) |  |
| --- | --- | --- | --- | --- |
| Universal primers | 1 | FY-J-214 | AAGCTHDTRGGTTCATAYCCY | Simon, 2006 |
|  |  | FY-N-1873 | AANGGDGGRTAVACNGTYCA | Simon, 2006 |
|  | 2 | FY-J-1423 | ACDAAYCAYAARGAYATYGG | Simon, 1994  et al., 1994 |
|  |  | FY-N-2329 | ACDGTRAAYATRTGRTGNGCYCA | Simon;1994 |
|  | 3 | FY-J-2198 | TATTHTGATTYTTYGGNCAYCCHGAAGT | Simon, 2006 |
|  |  | FY-N-3705 | GCYCCRCARATTTCNGAACATTG | Simon, 2006 |
|  | 4 | FY-J-4463 | TTYGCHCAYYTDGTNCCNCARGG | Simon, 2006 |
|  |  | FY-N-5748 | GGRTCRAANCCRCAYTCRAANGG | Simon, 2006 |
|  | 5 | FY-J-5747 | CCATTYGAATGTGGATTTGAYCC | Simon, 2006 |
|  |  | FY-N-6160 | YCAATTMTATCATTAACAGTGA | Simon, 2006 |
|  | 6 | FY-J-7077 | AARTCCTTWGARTAAAAKCC | Simon, 2006 |
|  |  | FY-N-7793 | TTRGGTWGRGATGGDTTRGG | Simon, 2006 |
|  | 7 | FY-J-7572 | AAANGGRATYTGNGCDCTYTTHGT | Simon, 2006 |
|  |  | FY-N-8741 | AYTTCRATNGYTTGHCCHT | Simon, 2006 |
|  | 8 | FY-J-8641 | CNGAHGAACAHARNCCRTG | Simon, 2006 |
|  |  | FY-N-9629 | GTHTGYGARGGAGCWYTKGG | Simon, 2006 |
|  | 9 | FY-J-10885 | AYGTYCTRCCYTGRGGWCARATRTC | Simon, 1994 |
|  |  | FY-N-12964 | TTACCTTARGGATAACAGCRTAW | Zhang et al., 2018 |
|  | 10 | FY-J-11335 | CAYATYCARCCHGARTGRTA | Zhang et al. 2008 |
|  |  | FY-N-12965 | TTACCTTAGGGATAACAGCRTWA | Zhang et al. 2018 |
|  | 11 | FY-J-12831 | CGGTYTGAACTCAGATCATGTA | Simon, 1994 |
|  |  | FY-N-13889 | KTACCTTKTGTATCAGGGTT | Simon, 2006 |
|  | 12 | FY-J-13286 | CTTTGCACRGTCAVWATACYGC | Zhang et al., 2018 |
|  |  | FY-14722 | GTGCCAGCVDCCGCGGTTANA | Simon, 2006 |
| Specific primers | 13 | HDJL-J-3337 | ATTGCTTTACCCTCCTTACG |  |
|  |  | HDJL-N-4447 | CCTTGTGGAACTAGGTGAGC |  |
|  | 14 | HDJL-J-3703 | CATTAGATGACTGAAAGCAAG |  |
|  |  | HDJL-N-7518 | AATCTTATCTATGGGGTATGC |  |
|  | 15 | HDJL-J-6797 | TAAATAAGCATACCCCATAG |  |
|  |  | HDJL-N-8176 | TATTACATTGGGTTCCTTTA |  |
|  | 16 | HDJL-J-9565 | AGCACCCTCACATACACTAA |  |
|  |  | HDJL-N-10988 | ATGGACTATTGTAGCAGCAG |  |
|  | 17 | HDJL-J-14119 | TAAAAAGAGTGACGGGCGA |  |
|  |  | HDJL-N- 454 | GTTGTGGATGCTAATGCTTG |  |
