# Supplementary material for: Characteristics of the complete mitochondrial genome of Suhpalacsa longialata (Neuroptera, Ascalaphidae) and its phylogenetic implications
Source: PeerJ. 2018 Nov 14;6:e5914. doi: 10.7717/peerj.5914 (PMC6240338; doi:10.7717/peerj.5914)
Supplement: Supplemental Information 2 [file peerj-06-5914-s002.doc]

**Table S2 Species used to construct the phylogenetic relationships along with GenBank accession numbers.**

| Order | Family | Species | GenBank accession number | | References | |
| --- | --- | --- | --- | --- | --- | --- |
| Neuroptera | Myrmeleontidae | *Myrmeleon immanis* | KM216750 | | Zhang & Wang., 2016 | |
|  |  | *Epacanthaclisis banksi* | KF701327 | | Cheng et al., 2015 | |
|  |  | *Gatzara jezoensis* | KY364372 | | Zhang & Yang, 2017 | |
|  |  | *Bullanga florida* | KX369241 | | Lan et al., 2016 | |
|  |  | *Dendroleon pantherinus* | KT425068 | | Wang et al., 2012 | |
|  | Chrysopidae | *Apochrysa matsumurae* | AP011624 | | Haruyama et al., 2011 | |
|  |  | *Chrysoperla nipponensis* | AP011623 | | Haruyama et al., 2011 | |
|  |  | *Chrysopa pallens* | JX033119 | | He et al., 2012 | |
|  |  | *Chrysoperla externa* | KU877169 | | Directly submitted | |
|  |  | *Nothochrysa* sp. | KP264630 | | Directly submitted | |
|  |  | *Nothancyla verreauxi* | KP264629 | | Directly submitted | |
|  |  | *Abachrysa eureka* | KY587199 | | Jiang et al., 2017 | |
|  | Ascalaphidae | *Ascalohybris subjacens* | KC758703 | | Cheng et al., 2014 | |
|  |  | *Ascaloptynx appendiculatus* | FJ171324 | Beckenbach & Steward, 2008 | |  |
|  |  | *Libelloides macaronius* | FR669150 | | Negrisolo et al., 2011 | |
|  |  | *Suhpalacsa longialata* | MH361300 | | This study | |
|  | Ithonidae | *Polystoechotes punctatus* | FJ171325 | | Beckenbach et al., 2008 | |
|  |  | *Oliarces clara* | KT425090 | | Wang et al., 2017 | |
|  |  | *Fontecilla graphicus* | KT425072 | | Wang et al., 2017 | |
|  | Hemerobiidae | *Neuronema laminatum* | KR078257 | | Zhao et al., 2016 | |
|  |  | *Drepanepteryx phalaenoides* | KT425087 | | Wang et al., 2017 | |
|  |  | *Micromus* sp. | KT425075 | | Wang et al., 2017 | |
|  | Osmylidae | *Thyridosmylus langii* | KC515397 | | Zhao et al., 2013 | |
|  |  | *Heterosmylus* sp. | KT425077 | | Wang et al., 2017 | |
|  | Mantispidae | *Ditaxis biseriata* | FJ859906 | | Cameron et al., 2009 | |
|  |  | *Eumantispa harmandi* | KT425080 | | Wang et al., 2017 | |
|  | Rapismatidae | *Rapisma zayuanum* | KF626447 | | Wang et al., 2013 | |
|  |  | *Rapisma xizangense* | KF626446 | | Wang et al., 2013 | |
|  | Psychopsidae | *Balmes birmanus* | KT425083 | | Wang et al., 2017 | |
|  |  | *Psychopsis coelivaga* | KT425082 | | Wang et al., 2017 | |
|  | Nemopteridae | *Chasmoptera huttii* | KT425069 | | Wang et al., 2017 | |
|  |  | *Nemoptera coa* | KT425079 | | Wang et al., 2017 | |
|  | Berothidae | *Podallea* sp. | KT425091 | | Wang et al., 2017 | |
|  |  | *Stenobiella* sp. | KT425081 | | Wang et al., 2017  / | |
|  | Sisyridae | *Climacia areolaris* | KT425088 | | Wang et al., 2017 | |
|  |  | *Sisyra nigra* | KT425070 | | Wang et al., 2017 | |
|  | Coniopterygidae | *Coniopteryx* sp. | KT425078 | | Wang et al., 2017 | |
|  |  | *Semidalis aleyrodiformis* | KT425067 | | Wang et al., 2017 | |
|  | Nevrorthidae | *Nipponeurorthus fuscinervis* | KT425076 | | Wang et al., 2017 | |
|  |  | *Nevrorthus apatelios* | KT425074 | | Wang et al., 2017 | |
|  | Nymphidae | *Nymphes myrmeleonoides* | KJ461322 | | Yan et al., 2014 | |
|  |  | *Myiodactylus osmyloides* | KT425089 | | Wang et al., 2017 | |
|  | Dilaridae | *Dilar* sp. | KT425073 | | Wang et al., 2017 | |
| Megaloptera | Corydalidae | *Corydalus cornutus* | FJ171323 | | Beckenbach et al., 2008 | |
|  |  | *Dysmicohermes ingens* | KJ806318 | | Wang et al., 2016 | |
|  |  | *Neochauliodes bowringi* | JQ351950 | | Li et al., 2015 | |
|  | Sialidae | *Sialis hamata* | FJ859905 | | Cameron et al., 2009 | |
