# Supplementary material for: Characteristics of the complete mitochondrial genome of Suhpalacsa longialata (Neuroptera, Ascalaphidae) and its phylogenetic implications
Source: PeerJ. 2018 Nov 14;6:e5914. doi: 10.7717/peerj.5914 (PMC6240338; doi:10.7717/peerj.5914)
Supplement: Supplemental Information 3 [file peerj-06-5914-s003.docx]

| Codon | Count | | | | RSCU | | | | Codon | Count | | | | RSCU | | | |
| --- | --- | --- | --- | --- | --- | --- | --- | --- | --- | --- | --- | --- | --- | --- | --- | --- | --- |
|  | SL | LM | AA | AS | SL | LM | AA | AS |  | SL | LM | AA | AS | SL | LM | AA | AS |
| UUU(F) | 264 | 261 | 279 | 271 | 1.59 | 1.58 | 1.67 | 1.62 | UAU(Y) | 150 | 141 | 151 | 167 | 1.62 | 1.58 | 1.59 | 1.72 |
| UUC(F) | 69 | 69 | 55 | 64 | 0.41 | 0.42 | 0.33 | 0.38 | UAC(Y) | 35 | 38 | 39 | 27 | 0.38 | 0.42 | 0.41 | 0.28 |
| UUA(L1) | 358 | 358 | 344 | 403 | 3.73 | 3.76 | 3.66 | 4.18 | CAU(H) | 56 | 55 | 58 | 61 | 1.44 | 1.45 | 1.55 | 1.56 |
| UUG(L1) | 71 | 42 | 62 | 39 | 0.74 | 0.44 | 0.66 | 0.4 | CAC(H) | 22 | 21 | 17 | 17 | 0.56 | 0.55 | 0.45 | 0.44 |
| CUU(L2) | 49 | 66 | 71 | 66 | 0.51 | 0.69 | 0.76 | 0.68 | CAA(Q) | 62 | 66 | 66 | 58 | 1.8 | 1.76 | 1.86 | 1.68 |
| CUC(L2) | 7 | 9 | 6 | 3 | 0.07 | 0.09 | 0.06 | 0.03 | CAG(Q) | 7 | 9 | 5 | 11 | 0.2 | 0.24 | 0.14 | 0.32 |
| CUA(L2) | 88 | 90 | 76 | 65 | 0.92 | 0.95 | 0.81 | 0.67 | AAU(N) | 147 | 153 | 155 | 156 | 1.58 | 1.69 | 1.68 | 1.71 |
| CUG(L2) | 3 | 6 | 5 | 3 | 0.03 | 0.06 | 0.05 | 0.03 | AAC(N) | 39 | 28 | 29 | 26 | 0.42 | 0.31 | 0.32 | 0.29 |
| AUU(I) | 296 | 309 | 339 | 307 | 1.7 | 1.73 | 1.81 | 1.69 | AAA(K) | 56 | 65 | 69 | 68 | 1.38 | 1.44 | 1.66 | 1.68 |
| AUC(I) | 52 | 49 | 36 | 56 | 0.3 | 0.27 | 0.19 | 0.31 | AAG(K) | 25 | 25 | 14 | 13 | 0.62 | 0.56 | 0.34 | 0.32 |
| AUA(M) | 212 | 225 | 208 | 233 | 1.69 | 1.76 | 1.79 | 1.81 | GAU(D) | 63 | 60 | 57 | 64 | 1.73 | 1.67 | 1.68 | 1.75 |
| AUG(M) | 39 | 31 | 24 | 25 | 0.31 | 0.24 | 0.21 | 0.19 | GAC(D) | 10 | 12 | 11 | 9 | 0.27 | 0.33 | 0.32 | 0.25 |
| GUU(V) | 102 | 89 | 105 | 83 | 2.05 | 1.77 | 2.14 | 1.78 | GAA(E) | 66 | 72 | 71 | 73 | 1.71 | 1.76 | 1.73 | 1.83 |
| GUC(V) | 5 | 6 | 2 | 4 | 0.1 | 0.12 | 0.04 | 0.09 | GAG(E) | 11 | 10 | 11 | 7 | 0.29 | 0.24 | 0.27 | 0.18 |
| GUA(V) | 82 | 95 | 85 | 90 | 1.65 | 1.89 | 1.73 | 1.94 | UGU(C) | 33 | 36 | 32 | 37 | 1.69 | 1.67 | 1.78 | 1.76 |
| GUG(V) | 10 | 11 | 4 | 9 | 0.2 | 0.22 | 0.08 | 0.19 | UGC(C) | 6 | 7 | 4 | 5 | 0.31 | 0.33 | 0.22 | 0.24 |
| UCU(S2) | 83 | 67 | 81 | 77 | 2.04 | 1.64 | 1.92 | 1.82 | UGA(W) | 82 | 83 | 89 | 87 | 1.71 | 1.66 | 1.76 | 1.78 |
| UCC(S2) | 18 | 13 | 8 | 17 | 0.44 | 0.32 | 0.19 | 0.4 | UGG(W) | 14 | 17 | 12 | 11 | 0.29 | 0.34 | 0.24 | 0.22 |
| UCA(S2) | 110 | 122 | 129 | 113 | 2.7 | 2.98 | 3.06 | 2.67 | CGU(R) | 27 | 20 | 28 | 21 | 1.86 | 1.48 | 1.96 | 1.5 |
| UCG(S2) | 0 | 4 | 1 | 3 | 0 | 0.10 | 0.02 | 0.07 | CGC(R) | 1 | 0 | 2 | 0 | 0.07 | 0 | 0.14 | 0 |
| CCU(P) | 67 | 52 | 63 | 59 | 2.02 | 1.59 | 1.94 | 1.79 | CGA(R) | 27 | 30 | 27 | 28 | 1.86 | 2.22 | 1.89 | 2 |
| CCC(P) | 14 | 13 | 11 | 16 | 0.42 | 0.40 | 0.34 | 0.48 | CGG(R) | 3 | 4 | 0 | 7 | 0.21 | 0.30 | 0 | 0.5 |
| CCA(P) | 51 | 63 | 55 | 55 | 1.53 | 1.92 | 1.69 | 1.67 | AGU(S1) | 44 | 32 | 40 | 42 | 1.08 | 0.78 | 0.95 | 0.99 |
| CCG(P) | 1 | 3 | 1 | 2 | 0.03 | 0.09 | 0.03 | 0.06 | AGC(S1) | 3 | 4 | 4 | 5 | 0.07 | 0.10 | 0.09 | 0.12 |
| ACU(T) | 85 | 73 | 91 | 70 | 1.62 | 1.47 | 1.75 | 1.45 | AGA(S1) | 68 | 85 | 73 | 80 | 1.67 | 2.08 | 1.73 | 1.89 |
| ACC(T) | 16 | 21 | 10 | 21 | 0.3 | 0.42 | 0.19 | 0.44 | AGG(S1) | 0 | 0 | 1 | 2 | 0 | 0 | 0.02 | 0.05 |
| ACA(T) | 107 | 101 | 104 | 96 | 2.04 | 2.04 | 2.00 | 1.99 | GGU(G) | 91 | 79 | 86 | 62 | 1.65 | 1.50 | 1.62 | 1.2 |
| ACG(T) | 2 | 3 | 3 | 6 | 0.04 | 0.06 | 0.06 | 0.12 | GGC(G) | 3 | 4 | 1 | 3 | 0.05 | 0.08 | 0.02 | 0.06 |
| GCU(A) | 95 | 78 | 78 | 78 | 2.17 | 1.72 | 1.77 | 1.82 | GGA(G) | 101 | 102 | 109 | 106 | 1.84 | 1.93 | 2.06 | 2.05 |
| GCC(A) | 12 | 21 | 21 | 22 | 0.27 | 0.46 | 0.48 | 0.51 | GGG(G) | 25 | 26 | 16 | 36 | 0.45 | 0.49 | 0.3 | 0.7 |
| GCA(A) | 68 | 79 | 72 | 69 | 1.55 | 1.75 | 1.64 | 1.61 |  |  |  |  |  |  |  |  |  |
| GCG(A) | 0 | 3 | 5 | 2 | 0 | 0.07 | 0.11 | 0.05 |  |  |  |  |  |  |  |  |  |

**Table S3 The codon number and relative synonymous codon usage (RSCU) in *S. longialata* mitochondrial protein coding genes.**

**Notes.**

SL, *S. longialata* (MH361300); LM, *L. macaronius*; AA, *A. appendiculatus*; AS, *A. subjacens*
